# Supplementary material for: A study on the influencing factors of the public’s willingness to donate funds for critical illness crowdfunding projects on network platforms
Source: PLoS One. 2022 Mar 11;17(3):e0263706. doi: 10.1371/journal.pone.0263706 (PMC8916639; doi:10.1371/journal.pone.0263706)
Supplement: Attachment — Submitted filename: Response to Reviewers.pdf [file pone.0263706.s001.pdf]

Response to Reviewers

Dear Editor and Reviewers,

Re: Manuscript **PONE-D-21-03866,** A study on the influencing factors of the public's willingness to donate funds for critical illness crowdfunding projects on network platforms.

Please find attached a revised version of our manuscript "PONE-D-21-03866", which we would like to resubmit for publication as a Original Research in PLoS ONE.

Your comments were highly insightful and enabled us to greatly improve the quality of our manuscript. In the following pages are our point-by-point responses to each of the comments of the reviewers as well as your own comments.
Revisions in the text are shown using red highlight for additions.

According to the recommendation of reviewer 1, 2 and 3, we will reply to the opinions of the three reviewers below, including what specific improvements we have made to the manuscript according to the opinions of the reviewers.

Similarly, after revising according to the reviewers' opinions, other parts of the full text are modified accordingly, such as the abstract, literature review and theoretical part, and a series of tables and figures are also included.

If there are still areas for improvement, we are willing to seriously revise them as soon as possible.

What needs to be added is that there is a commen in the review comments that our English expression is not standardized, but it needs to be explained. When we submitted the first manuscript, we already chose the editing agency suggested by PLoS and the manuscript was edited by one or more of the highly qualified native English speaking editors at AJE. And attach a editing certificate. We don't know which part of the problem led to such comment.

Once again, we sincerely thank you for your enthusiastic work, your careful evaluation and patient guidance. We learned a lot. It was a very pleasant experience.

AJE

Editing Certificate

This document certifies that the manuscript

A Study on the Influencing Factors of Public's Willingness to Donate Funds for Serious Illness Crowdfunding Projects on the Network Platform

prepared by the authors

Lu Chen, Fan Luo, Wanshi He, Heng Zhao and Liru Pan

was edited for proper English language, grammar, punctuation, spelling, and overall style by one or more of the highly qualified native English speaking editors at AJE.

This certificate was issued on **January 29, 2021** and may be verified on the AJE website using the verification code **6E2C-BF80-DDA1-6D86-6BCP**.

Neither the research content nor the authors' intentions were altered in any way during the editing process. Documents receiving this certification should be English-ready for publication; however, the author has the ability to accept or reject our suggestions and changes. To verify the final AJE edited version, please visit our verification page at aje.com/certificate. If you have any questions or concerns about this edited document, please contact AJE at support@aje.com.

AJE provides a range of editing, translation, and manuscript services for researchers and publishers around the world. For more information about our company, services, and partner discounts, please visit aje.com.

We hope that the revision of the manuscript and our reply will be enough to make our manuscript suitable for publication in PLoS ONE.

We look forward to hearing from you at your earliest convenience.

Yours sincerely,
Lu Chen

CATALOGUE

Response to Reviewer #1 3
Response to Reviewer #2 6
Response to Reviewer #3 9
Response to Journal Requirements 12

Reviewer #1:

Manuscript PONE-D-21-03866 is a study of the influencing factors of the public willingness to donate funds to critical illness via crowd-funding platforms.
They use a well established framework of structural equation modeling (SEM) evalute path coefficient for 14 well grounded hypotheses.
1) The manuscript would benefit from exploratory data analysis (visual). For example, it would be illustrative to see the distributions of potential variables to confirm their "normality". Of course in this particular case the variables are categorigal and SEM can be used only approximately, which should mentioned in the analysis.Another example would be visualing correlation between varios external variables (gender, age and income) and potential variables.

2) It would be great to evaluating the effect of gender, age and income level on the path coefficients. What if there is significant divergence there? For example if between two groups one has positive effect and the other negative, the overall result could be close to zero, as in H1.
Overall after a minor revision the manuscript is suitable for publication.

Our replies:

Thank you very much for your careful review and constructive suggestions with regard to our manuscript.
Thoses comments are very helpful for us to revise and improve our paper.

There are two main aspects of reviewer 1's comments, which are to be answered one by one.

1. For the first suggestion, we have observed the distributions of potential variables and determined that they are all approximately "normality". Of course, in this special case, variables are classified, SEM can only be used approximately, We also emphasized this sentence in the revised manuscript（The details are shown from line 503~505）

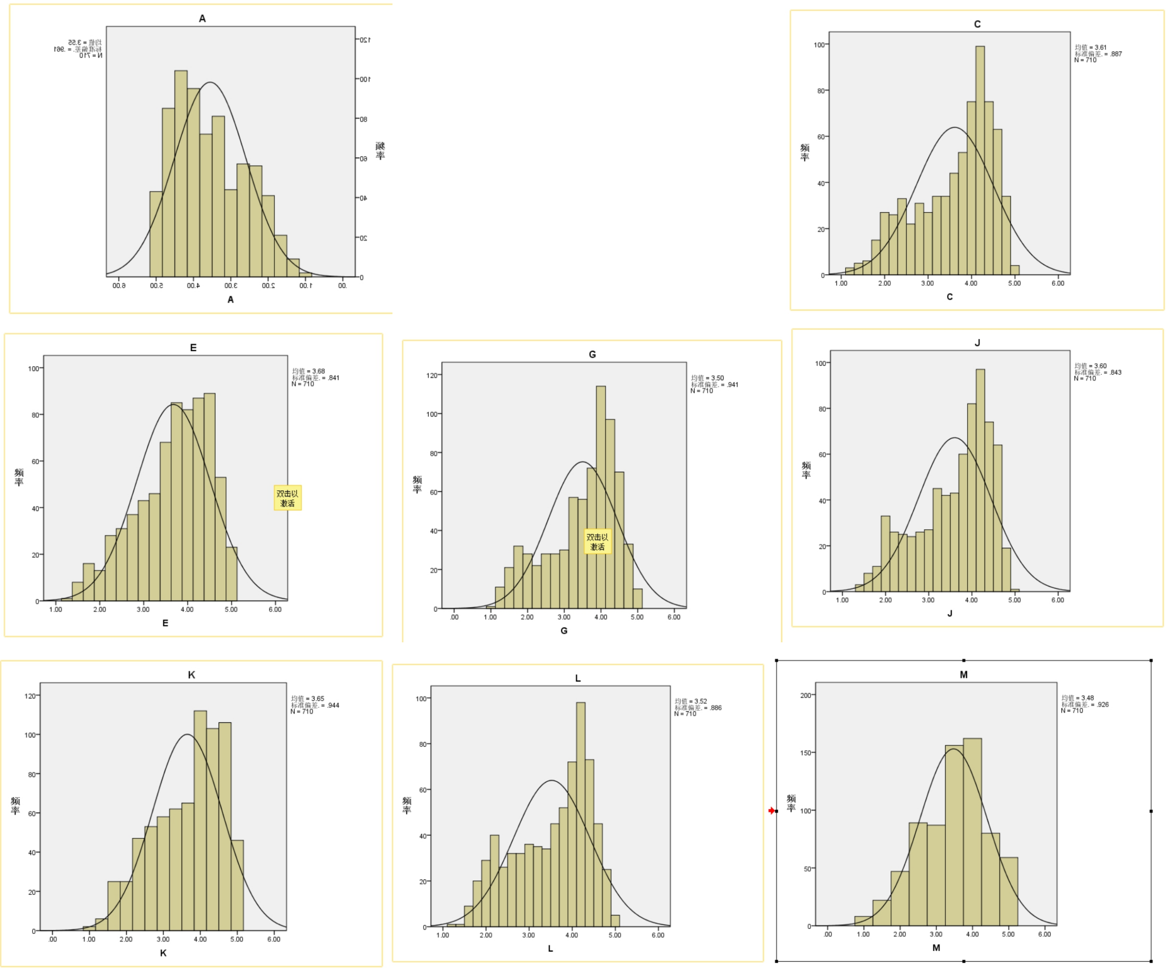

A— Donation attitude；C— Empathic Concern；E— Perceived usefulness；G— Platform trust；

J— Interactivity；k— Donation willingness；L— Social distance；M— Donation behavior

2. For the second question, we have evaluated the effects of gender, age and income level on the path coefficients. Through grouping observation, We find that there is no significant difference in the overall model path through grouping observation, and some paths, such as men, are negative, but not significant. The above reasons are due to the differences of

samples, that is, there are differences in cognition and behavior among different groups.

We hope that the corrections will meet with your concerns.
Special thanks to you for your good comments.

Reviewer #2:

This is obviously an interesting article. However, there are few issues that need to looked at.

Find the following:

1. For example, the article mentions both "Internet +" donations and "Internet donations". How different are these two concepts?
2. Besides, it would have been helpful especially for readers who may not be conversant with this model of donation to know exactly what "Internet +" is. Is it generally known or it's restricted to only the study area?
3. It may be helpful to provide distinctions among the 12 latent variables that were considered. How different are they from each other?
4. It was also not to clear the target of the recommendations. The suggestion is for the recommendations to be targeted at particular person/institution etc.

Our replies:

Thank you very much for your careful review and constructive suggestions with regard to our manuscript. Thoses comments are very helpful for us to revise and improve our paper. There are three main aspects of reviewer 2's comments, which are to be answered one by one.

1. **The first and second questions you mentioned, especially about the articles both "Internet +" donations and "Internet donations",** we also found this kind of problems when we checked them. There is no obvious difference between the two, but it does cause ambiguity to readers. In the revised manuscript, there has been a unified way of expression, namely "Internet donations". Thank you for your advice.

2. There are mainly eight variables in the revised and improved model. No matter the previous 12 variables or the current 8 variables, they are mainly based on the two classic models in the field of consumer behavior research——TAM and TPB.

The reason is that TAM can explore the trade-off process when the public uses the platform as a system function. As a general model, TAM only provides two cognitive concepts that affect individuals' willingness to accept technology, namely perceived usefulness and perceived ease of use. It does not explain or limit the external variables that affect the usefulness and ease of use in specific application situations, while TPB combines various variables, including the public's attitude towards behavior and the influence of others.

Therefore, in the TAM model, in order to better explain the public's willingness to donate and decision-making behavior under the specific circumstances of the critical illness crowdfunding platform, the public's **trust in the platform** is taken as an important premise of perceived usefulness, which is based on the design of the **interactive** mechanism provided by the platform.

TAM model itself includes four variables: **perceived usefulness, donation attitude, donation willingness and donation behavior.**

Personal empathy is also added as a variable of personal factors to measure the public's willingness to donate to the crowdfunding projects for major diseases on the Internet platform.

In addition, in the part of TPB model, the TPB holds that the behavior of an individual is not always controlled by his or her own will. Rather, the individual's behavior is affected by internal and external factors. Attitudes and standardized responses to performed situations will change the individual's behavior, so we added the variable of **social distance.**

Therefore, there are eight variables.

（The details are shown from line 108~372）

3. For the fourth question, thank you very much for your suggestions, including your suggestion that the suggestion is for the recommendations to be targeted at particular person / institution, etc

We have readjusted this part according to your suggestions, which can be divided into suggestions for the government, platforms and help-seekers.

（The details are shown from line 611~708）

We hope that the corrections will meet with your concerns.
Special thanks to you for your good comments.

Reviewer #3:

The authors are quite ambitious to use 45 variables with 12 latent factors in a SEM (n = 710) to explain the behaviour related to crowdfunding projects on network platform in China. However, the proposed model failed to fulfil the minimum criteria for adequate model fit, with merely GFI = 0.788, CFI = 0.835 and X2/df = 3.909 (p. 35-36), i.e. the entire manuscript needs to be re-written, with revised research questions,
hypothesis, theoretical framework, and data analysis.

More importantly, the theoretical framework is very weak, the authors only list out various theories and perspectives (p. 12-23) but without showing any linkage with reference to the existing literature.
The conclusions and recommendations, such as urging for more governmental supervision, by using legal framework and blockchain technology for further government monitoring etc., did not based on the results/empirical findings.
Hence, I do not think the current form of manuscript is suitable for publication. There are major flaws in the research design and data analysis methods. There is also lack of theoretical contribution.

Our replies:

Thank you very much for your careful review and constructive suggestions with regard to our manuscript. Thoses comments are very helpful for us to revise and improve our paper. There are three main aspects of reviewer 3's comments, which are to be answered one by one.

1. **The first question** is that the proposed model does not meet the fitting standard.

With a little explanation of this situation, in the first draft, there were 12 variables considered at that time, and the whole model was more complex. Therefore, the requirements for individual index data were lowered, only close to 0.9, but not more than 0.9. It was really worth reflecting that they were reported as model fitting results.
To solve this problem, in this draft, our revision ideas and specific measures are as follows:
Reorganize variables and models. In the original TAM model, a few variables, such as perceived ease of use, which are not suitable for current situation discussion, are eliminated.

In traditional TAM, the higher the ease of use of a critical illness crowdfunding platform perceived by the public is, the higher the usefulness and effectiveness of the platform perceived by the public. However, the ease of use of the Internet platform has been very high after years of development, So the variable of the ease of use will not be considered in this study. At the same time, considering that both subjective norm and social distance measure the influence of the surrounding groups on the donors, we choose the variable of social distance in the new draft based on the theoretical basis and practical observation. To sum up, there are 8 variables remaining in the model and 8 hypotheses proposed. The model fits well and all the path hypotheses are valid.

（The details are shown from line 512~517）

2. **The second question** is about the insufficient theory.

In the process of revising the manuscript, we still rescreened the relevant literature, but the relevant literature is still not rich.

(1) There are few empirical studies on this topic in China, and there are few high-level literatures;

(2) Foreign related literature is not much, some donation articles mainly focus on organ donation;

(3) Although there are not many high-level literatures for reference, **it is still a social phenomenon and hot issue of great research significance.** Therefore, we start from the perspective of consumer behavior and choose the classic TAM and TPB models. The variables commonly used in these two models are relatively fixed, and the literatures on these two models are very common, Therefore, there is not too much theoretical elaboration in the article, and the article introduces the theory and literature basis of path hypothesis in detail. As far as the current structure is concerned, both the literature review and the path hypothesis are fairly long. If the reviewers feel that it is still necessary to explain the TAM and TPB variables in detail, we will complete it as soon as possible.

3. **The third question** is about policy recommendations. In this draft, we made the following adjustments:

(1) Readjust the structure and level of the proposal part. It can be divided into for government, platforms and help-seekers;

(2) As for the government's proposal you mentioned, which has no basis, we have also

made an explanation and response. The proposal to strengthen government supervision, such as improving legislation, is based on the review of the current laws and government policy reports. The reason why we didn't launch the discussion is that the theme of the discussion is the influencing factors and paths of donation behavior. Of course, if we all agree that we should consolidate here, we will continue to work hard.

We hope that the corrections will meet with your concerns.
Special thanks to you for your good comments.

Response to Journal Requirements

Dear Editor,

I'm very glad to receive your email. It's very nice of you to proofread our manuscript. The following part is about some of the necessity of Journal Requirements.

According to your suggestions, we have responded one by one and adjusted the corresponding parts in the revised manuscript, and our replies are marked in yellow.

Thank you again for your hard work.

If there is anything wrong, please let me know.

Thank you.

When submitting your revision, we need you to address these additional requirements.

1. Please ensure that your manuscript meets PLOS ONE's style requirements, including those for file naming.

Thank you for your suggestion. The revised manuscript has been received according to the style requirements of PLoS One to arrange the chapters and layout. If there is anything wrong, we will improve it according to the requirements of the journal.

2. Please include additional information regarding the survey or questionnaire used in the study and ensure that you have provided sufficient details that others could replicate the analyses. For instance, if you developed a questionnaire as part of this study and it is not under a copyright more restrictive than CC-BY, please include a copy, in both the original language and English, as Supporting Information.

Thank you for your reminding. We have provided enough details about the survey or questionnaire used in the study in Table 1, and others can copy our questionnaire and analyze it.

3. We note that you have indicated that data from this study are available upon request. PLOS only allows data to be available upon request if there are legal or ethical restrictions on sharing data publicly. For more information on unacceptable data access restrictions, please see http://journals.plos.org/plosone/s/data-availability#loc-unacceptable-data-access-restrictions.

In your revised cover letter, please address the following prompts:
a) If there are ethical or legal restrictions on sharing a de-identified data set, please explain them in detail
(e.g., data contain potentially sensitive information, data are owned by a third-party organization, etc.) and
who has imposed them (e.g., an ethics committee). Please also provide contact information for a data access
committee, ethics committee, or other institutional body to which data requests may be sent.

b) If there are no restrictions, please upload the minimal anonymized data set necessary to replicate your
study findings as either Supporting Information files or to a stable, public repository and provide us with the
relevant URLs, DOIs, or accession numbers. For a list of acceptable repositories, please see
http://journals.plos.org/plosone/s/data-availability#loc-recommended-repositories.
We will update your Data Availability statement on your behalf to reflect the information you provide.

We have uploaded the minimum anonymous data set, which includes the values behind the reported average, standard deviation and other measures, as well as the values used to build the graph(in **Table 1 ~ Table 5**）

4. PLOS requires an ORCID iD for the corresponding author in Editorial Manager on papers submitted after
December 6th, 2016. Please ensure that you have an ORCID iD and that it is validated in Editorial Manager. To
do this, go to 'Update my Information' (in the upper left-hand corner of the main menu), and click on the
Fetch/Validate link next to the ORCID field. This will take you to the ORCID site and allow you to create a new
iD or authenticate a pre-existing iD in Editorial Manager. Please see the following video for instructions on
linking an ORCID iD to your Editorial Manager account:
https://www.youtube.com/watch?v=_xcclfuvtxQ

Thank you for your reminding. Our three corresponding authors have updated their ORCID ID, which are:

Fan Luo https://orcid.org/0000-0002-8487-0200

Wanshi He https://orcid.org/0000-0001-8109-6566

Heng Zhao https://orcid.org/0000-0002-7344-4329

5. We note that Figure 3 in your submission contain map images which may be copyrighted.

All PLOS content is published under the Creative Commons Attribution License (CC BY 4.0), which means that the manuscript, images, and Supporting Information files will be freely available online, and any third party is permitted to access, download, copy, distribute, and use these materials in any way, even commercially, with proper attribution. For these reasons, we cannot publish previously copyrighted maps or satellite images created using proprietary data, such as Google software (Google Maps, Street View, and Earth). For more information, see our copyright guidelines: http://journals.plos.org/plosone/s/licenses-and-copyright.

Thank you for your reminding. Considering that the figure is not important to the full text, so we deleted the figure in the revised manuscript.
