# Supplementary material for: A study on the influencing factors of the public’s willingness to donate funds for critical illness crowdfunding projects on network platforms
Source: PLoS One. 2022 Mar 11;17(3):e0263706. doi: 10.1371/journal.pone.0263706 (PMC8916639; doi:10.1371/journal.pone.0263706)
Supplement: Attachment — Submitted filename: Response to Reviewers.pdf [file pone.0263706.s002.pdf]

Response to Reviewers

Dear Editor and Reviewers,

Re: Manuscript PONE-D-21-03866R1, A study on the influencing factors of the public's willingness to donate funds for critical illness crowdfunding projects on network platforms.

Please find attached a revised version of our manuscript “PONE-D-21-03866R1”, which we would like to resubmit for publication as a Original Research in PLoS ONE.

Your comments were highly insightful and enabled us to greatly improve the quality of our manuscript. In the following pages are our point-by-point responses to each of the comments of the reviewers as well as your own comments.
Revisions in the text are shown using red highlight for additions.

First of all, thank you very much for the comments of the editor and reviewers. We have also made significant revisions to the paper. Now we will give a brief description of the work we have done, and then answer the comments of reviewers one by one in the second part.

1. In this part, nearly 20 new literatures have been added. Of course, necessary adjustments have been made to the logical expression. In the revised version with a trajectory, it is marked in red.

2. In the demonstration part, this draft is clearly divided into the EFA part and the CFA part, and relevant tables and figures are supplemented.

(1) Some necessary analyses were added to the Validity Test section. Skewness and kurtosis can effectively describe the normal distribution of data for each observed variable. The values of Skewness and kurtosis are shown in Table 3 within ±1. The absolute value of each skewness is less than 3, and the absolute value of each kurtosis is less than 10. All variables and averages are in accordance with the standard, which indicates the data shows a normal distribution.

(2) Secondly, Factor analysis in "Table 4. Rotated factor Analysis (factor analysis)". As can be seen from Table 4, the exploratory factor loads of 31 indicators were obtained through the maximum variance rotation analysis. These quantities are all greater than 0.5, satisfying the model requirements and allowing the extraction of 8 factors to explain the structure of the variables.

(3) The factor analysis of Confirmatory was improved. See Figure 3 starting at line 610 for details.

According to the suggestions, we have drawn detailed Path Coefficients of the Hypothetical Model. See Fig.4 in line 639.

3. Delete all recommendations that are not based on the study's findings, and make necessary streamlining of the summary, introduction and conclusion according to the adjustment of the main body.

The following are the responses to the suggestions of reviewers respectively:

Reviewer #1:

The authors did not adequately answer questions 1 and 2 raised previously.

1) On 137 the authors plot the distribution and claim that they observe that they are normal. It is customary to use a KS or D'Agostino and Pearson's test to test whether a distribution is normal. It clear from the figures most of the distributions are not normal, which invalidates the chosen approach. The second part regarding the visualization of interdependence of available features has been ignored.

2) The response to the second question is also verbal. No figures or metrics were provided. Quantitative estimates of 1) and 2) should appear in the main text, if they were made.
I conclude that the manuscript has not been brought to the requested level of clarity. A major revision is necessary.

Response:

Thanks for the reviewer's comments. On behalf of the team, I would like to apologize for our negligence in the last draft. As for the two problems you mentioned, we have carefully discussed and revised them in this draft.

1. The first question has been reflected in the text of this draft, for details, see 2 (1) in the answer section.

Some necessary analyses were added to the Validity Test section. Skewness and kurtosis can effectively describe the normal distribution of data for each observed variable. The values of Skewness and kurtosis are shown in Table 3 within ±1. The absolute value of each skewness is less than 3, and the absolute value of each kurtosis is less than 10. All variables and averages are in accordance with the standard, which indicates the data shows a normal distribution.

2. The second question is whether gender, age and income have a significant effect on the path coefficient.

The process of this analysis is not reflected in the current manuscript, but we have verified it. If necessary, we are also very happy to supplement the manuscript as soon as possible. The idea is as follows:

Divergence: We divided gender into men and women, aged under 30 years old and over 30 years old, and income into under 5000 yuan and over 5000 yuan, and then looked at the path coefficient respectively, but there was no significant difference in general. The age and income division takes into account the actual situation of the respondents, including the old Chinese saying "one should stand firm at the age of 30" and the average per capita income level of Chinese cities.

One path coefficient of gender is -0.071, which can be explained by the fact that women are more likely to have empathy than men when they want to donate, which is also consistent with some literature studies [1]. Sisco and Weber analyzed the influencing factors of online donation behavior on the GoFundMe platform, and their survey found that women are more likely to resonate with donation information [2].

The income path coefficient of -0.024 can be explained as well-off donors are more likely to make donations, which is also consistent with some literature studies [3-5].

Thanks again for the reviewer's comments, which are of great help to us. We also hope that our revision this time is consistent with your suggestions.

Gender

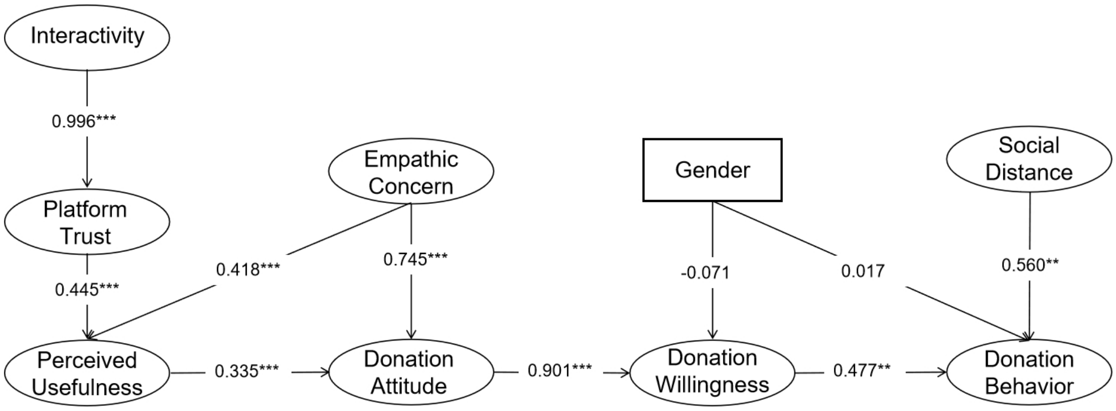

Fig 1.

Age

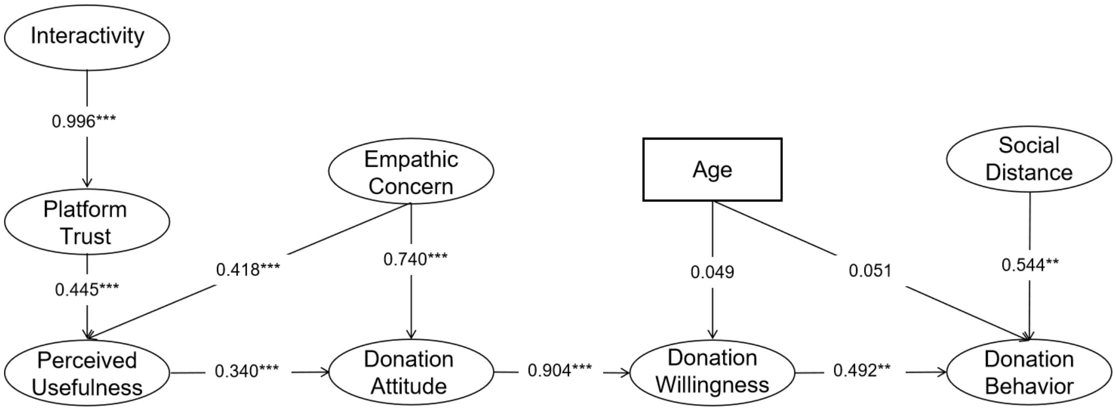

Fig 2.

Income

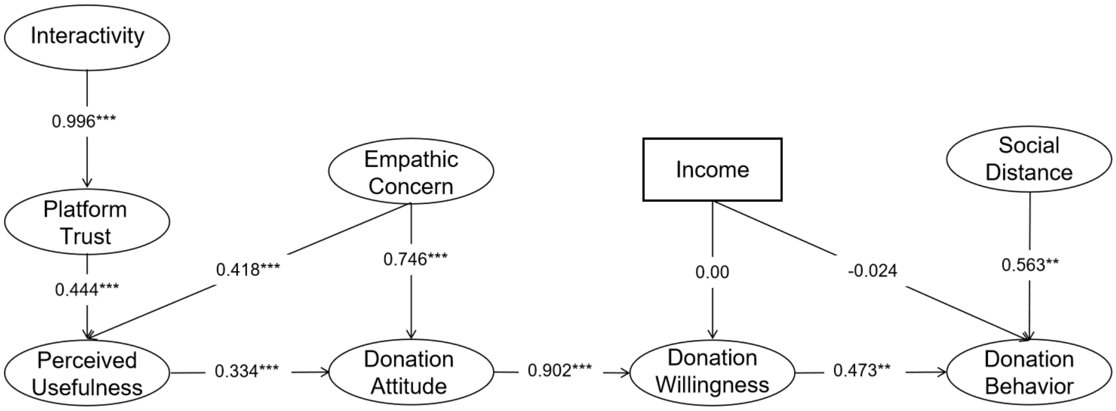

Fig 3.

Reference:

[1] Mesch,D.J., Brown,M.S, Moore,Z.l,& Hayat,A.D.(2011). Gender differences in charitable giving. International Journal of Nonprofit and Voluntary Sector Marketing,16(4),342-355. doi:10.1002/nvsm.432

[2] Sisco MR, Weber EU. Examining charitable giving in real-world online donations. Nat Commun. 2019;10: 3968.

[3] Schwienbacher A, Larralde B. Crowdfunding of small entrepreneurial ventures. In: Cumming D, editor. The Oxford handbook of entrepreneurial finance. Oxford, UK: Oxford University Press; 2012. pp. 369-391.

[4] Lin Z, Xiao Q, Zhou Z. An empirical study on the relationship between ethical predispositions and charitable behavior: Based on the moderating effect of moral identity. Foreign Econ Manag. 2014;36: 16-31.

[5] Hui J, Gerber E, Greenberg M. Easy money? The demands of crowdfunding work. Segal technical report. 1st ed. Evanston, IL: Northwestern University; 2012.

Reviewer #3:

However, the authors still failed to fully address my comments in point number 1, 2 and 3:

1) With regarded to the first point, the SEM result is now fulfilled the criteria for model fit, however, please provide more details about the estimator used (Li, 2016)? Did the model with any error terms correlated (Hermida, 2015)?

2) The discussion of the theoretical framework is still very thin, in addition to the TAM and TPB, the authors still need to provide theoretical justifications for including other variable in the model, such as empathy, trust, planning behavior and social distance. Developing a 'model' without much theoretical foundations are risky and dangerous, see Burghardt and Bodansky (2021) and Borsboom, van der Maas, Dalege, Kievit, and Haig (2021), and (DeYoung & Krueger, 2020).

3) As the authors mentioned, the proposal to strength government supervision was merely

'based on the review of the current laws and government policy reports'. Any recommendations that is not based on the study's findings should be removed to avoid any confusions to the reader.

Response:

Thank you very much for the reviewer's patient guidance. As for the three suggestions you mentioned, I would like to review our revision work on behalf of the team.

1. For the first question, provide more details about the estimator used.

We have completed drawing detailed Path Coefficients of the Hypothetical Model. See Fig.4 in line 639. Thank you.

2. Second question, thank you for your reminding.We strongly agree with your suggestions. On this issue, we are also very discreet. On the one hand, on the basis of the literature part before, we have collected the latest literature and omissions to enhance the theoretical basis, nearly more than 20 new references. See the red section for specific new content. On the other hand, we have carried on the exploratory factor analysis, through the principal component analysis ie. and a series of measures to ensure that the model construction is "mathematically" correct .For the third problem, this is a good suggestion. In this draft, all recommendations that are not based on the study's findings are deleted, and after the adjustment in the main body, the summary, introduction and conclusion are all simplified accordingly.

If there are still areas for improvement, we are willing to seriously revise them as soon as possible.

Once again, we sincerely thank you for your enthusiastic work, your careful evaluation and patient guidance. We learned a lot. It was a very pleasant experience.

We hope that the revision of the manuscript and our reply will be enough to make our manuscript suitable for publication in PLoS ONE.

We look forward to hearing from you at your earliest convenience.

Yours sincerely,

Lu Chen
