# Supplementary material for: A study on the influencing factors of the public’s willingness to donate funds for critical illness crowdfunding projects on network platforms
Source: PLoS One. 2022 Mar 11;17(3):e0263706. doi: 10.1371/journal.pone.0263706 (PMC8916639; doi:10.1371/journal.pone.0263706)
Supplement: Attachment — Submitted filename: Response to Reviewers.pdf [file pone.0263706.s003.pdf]

Response to Reviewers

Dear Editor and Reviewers,

Re: Manuscript PONE-D-21-03866R2, A study on the influencing factors of the public's willingness to donate funds for critical illness crowdfunding projects on network platforms.

Please find attached a revised version of our manuscript "PONE-D-21-03866R2", which we would like to resubmit for publication as a Original Research in PLoS ONE.

Your comments were highly insightful and enabled us to greatly improve the quality of our manuscript. In the following pages are our point-by-point responses to each of the comments of the reviewers as well as your own comments.
Revisions in the text are shown using red highlight and track changes for additions.

First of all, thank you very much for the comments of the editor and reviewers. We have also made significant revisions to the paper. Now we will answer the comments of reviewers one by one in the second part.

The following are the responses to the suggestions of reviewers respectively:

Reviewer #1:

The normality assumption had not been addressed properly.
I have already suggested to D'Agostino test for normality that yields a p-value that had been ignored.

"Skewness and kurtosis can effectively describe the normal distribution of data for each observed variable". - a very odd statement. Which is not followed by any statistical claim.

Response:

Thanks for the reviewer's comments.
As for your suggestions, we have carefully considered them in the last manuscript revision, including the D 'Agostino test of normal distribution you mentioned. In our last manuscript, we used "Skewness and Kurtosis" to check the normal distribution for the following two reasons.
First, we checked the journal Jiang W, Huang Z, Peng Y, Fang Y, Cao Y published in PLoS ONE (2020) Factors affecting Prefabricated construction Promotion in China: A

structural equation modeling. PLoS ONE 15(1): E0227787.
In his paper, the method of "Skewness and Kurtosis "was also adopted and specific references were given (No. 97 reference in this manuscript).
At the same time, in order to further confirm whether this method is a good method to verify the normal distribution, we also refer to literatures with up to 315 citations: Hopkins K D, Weeks D L. Tests for Normality and Measures of Skewness and Kurtosis:Their place in research reporting[J]. Educational and Psychological Measurement, 1990, 50(4): 717-729. (No. 98 reference in this manuscript)
In this paper Page 721 mentioned " In addition to an omnibus test of normality,separate measures and tests of skewness and kurtosis have descriptive and inferential value. (Some statisticians recommend that separate inferential tests for skewness and kurtosis be used only after an omnibus test has rejected the normality assumption, but Monte Carlo studies have demonstrated that with certain types of distributions the separate tests(eqs. 5 and 8)have greater power than even the Shapiro-wilk test(Shapiro et al., 1968).)"
Based on the above reasons, we've completed the normal distribution test follow the experience of papers published in the same journal(PLoS ONE) in the manuscript.

Reviewer #3:

Thanks for submitting the revised manuscript to further address my concerns. But there are still two outstanding issues need to be addressed and also related to my previous comments:
1) It seems the authors are using the CFA goodness-of-fit results (Figure 3, p. 29 and Table 6, p. 31) to justify the SEM (Figure 4, p. 32) also fulfil the criteria of adequate model fit. The authors said that 'As shown in Table 6, the same indices can be used to evaluate of both the CFA and SEM' (p. 32, lines 646-647). I am afraid that there is a serious methodological flaw here. The proposed SEM model and the sequences results (Table 7) cannot be accepted, unless the authors can show the SEM results' fit indices of Figure 4. The authors should also submit the anonymous raw data and syntax used to enable to readers to replicate the results.

2) For the discussion section, the authors should further discuss the major findings according to the existing theory and the literature. The entire section (p. 35-37) without any literature to support the discussion is a bit lack of scientific rigor.

Response:

Thanks for the reviewer's comments. Thank you for your seriously review. In view of your suggestions, we carefully check the full manuscript and calculation process again. The solution to the first suggestion is as follows:

In Table 6. The result of "Research Model" in the third line of the last manuscript was the fitting result of SEM model. Here we did not write clearly, and caused ambiguity, deeply ashamed. We have adjusted the form and text.

You mentioned lines 646-647 of the previous manuscript "As shown in Table 6, the same indices can be used to evaluate adequacy of both the CFA and SEM. All indices are in the appropriate range, indicating the model is acceptable. " I'm sorry for the confusion caused by the expression and writing. We didn't mean that the result of CFA was used to represent the result of SEM.

It was probably a misunderstanding caused by the unclear expression and identification of Table 6.The indicators observed by CFA and SEM are the same indicators, but the measurement results of the indicators are different. The indicator results of SEM and CFA meet the fitting requirements.As for the second suggestion you mentioned, we have submitted it to the designated website and provided a link to download the source data as required by PLoS. Thank you for your advice.

And thank you for your 3rd suggestions. We have completed them one by one and added some references. Thank you for your careful guidance, and we will pay more attention in the future.

If there are still areas for improvement, we are willing to seriously revise them as soon as possible.

Once again, we sincerely thank you for your enthusiastic work, your careful evaluation and patient guidance. We learned a lot. It was a very pleasant experience.

We hope that the revision of the manuscript and our reply will be enough to make our manuscript suitable for publication in PLoS ONE.

We look forward to hearing from you at your earliest convenience.

Yours sincerely,

Lu Chen
